# Evidence for the interaction of the human metapneumovirus G and F proteins during virus-like particle formation

**DOI:** 10.1186/1743-422X-10-294

**Published:** 2013-09-25

**Authors:** Liat Hui Loo, Muhammad Raihan Jumat, Yi Fu, Teck Choon Ayi, Pui San Wong, Nancy WS Tee, Boon Huan Tan, Richard J Sugrue

**Affiliations:** 1 Division of Molecular Genetics and Cell Biology, School of Biological Sciences, Nanyang Technological University, 60 Nanyang Drive, Singapore 637551, Singapore; 2 Microbiology Laboratory, KK Women’s and Children’s Hospital, 100 Bukit Timah Road, Singapore 229899, Singapore; 3 Detection and Diagnostics Laboratory, Defence Medical and Environmental Research Institute, DSO National Laboratories, 27 Medical Drive #13-00, Singapore 117510, Singapore

**Keywords:** Human metapneumovirus, Virus-like particle, Recombinant expression, HMPV F protein, HMPV G protein, Protein interactions

## Abstract

**Background:**

Human metapneumovirus (HMPV) is now a major cause of lower respiratory infection in children. Although primary isolation of HMPV has been achieved in several different cell lines, the low level of virus replication and the subsequent recovery of low levels of infectious HMPV have hampered biochemical studies on the virus. These experimental methodologies usually require higher levels of biological material that can be achieved following HMPV infection. In this study we demonstrate that expression of the HMPV F, G and M proteins in mammalian cells leads to HMPV virus-like particles (VLP) formation. This experimental strategy will serve as a model system to allow the process of HMPV virus assembly to be examined.

**Methods:**

The HMPV F, G and M proteins were expressed in mammalian cell lines. Protein cross-linking studies, sucrose gradient centrifugation and *in situ* imaging was used to examine interactions between the virus proteins. VLP formation was examined using sucrose density gradient centrifugation and electron microscopy analysis.

**Results:**

Analysis of cells co-expressing the F, G and M proteins demonstrated that these proteins interacted. Furthermore, in cells co-expression the three HMPV proteins the formation VLPs was observed. Image analysis revealed the VLPs had a similar morphology to the filamentous virus morphology that we observed on HMPV-infected cells. The capacity of each protein to initiate VLP formation was examined using a VLP formation assay. Individual expression of each virus protein showed that the G protein was able to form VLPs in the absence of the other virus proteins. Furthermore, co-expression of the G protein with either the M or F proteins facilitated their incorporation into the VLP fraction.

**Conclusion:**

Co-expression of the F, G and M proteins leads to the formation of VLPs, and that incorporation of the F and M proteins into VLPs is facilitated by their interaction with the G protein. Our data suggests that the G protein plays a central role in VLP formation, and further suggests that the G protein may also play a role in the recruitment of the F and M proteins to sites of virus particle formation during HMPV infection.

## Introduction

Human metapneumovirus (HMPV) is a new member of *Paramyxoviridae* that was first identified in children with respiratory diseases in Netherlands [1]. The clinical symptoms that are caused by HMPV infections in children are similar to those observed with respiratory syncytial virus (RSV) infection; ranging from mild symptoms to pneumonia. HMPV is now a globally recognised cause of lower respiratory infection in children [2,3]). Genetic analysis identified two major genogroups A and B [4-6]. HMPV expresses two major integral membrane proteins that play a role in virus entry. The attachment (G) protein plays a role in virus attachment and is expressed as a single polypeptide chain, which subsequently undergoes extensive N- and O-linked glycosylation [7]. The fusion (F) protein mediates fusion of the virus and host-cell membranes, and is initially synthesised as a single polypeptide chain (F0) that undergoes proteolytic cleavage to generate the mature and active form of the protein, consisting of F1 and F2 protein subunits [8]. The virus also expresses a third membrane-associated protein called the matrix (M) protein, which is analogous to the M protein of RSV and is a major determinant of virus morphology [9].

Primary isolation of HMPV has been achieved in several different cell lines [4,10,11], however tissue culture adapted isolates can require up to 21 days incubation before cytopathic effects are visualised [7,11-13]. This low level of virus replication and the subsequent recovery of low levels of infectious HMPV in standard cell culture have hampered biochemical studies on the virus. These experimental methodologies usually require higher levels of biological material than can be achieved following HMPV infection.

Virus-like particle (VLP) formation following the co-expression of specific virus structural proteins has been demonstrated in several paramyxoviruses [14-18]. These studies have allowed the identification of essential virus proteins that are required for virus particle assembly. Although a central role for the M protein in VLP formation has been reported for human parainfluenza type 1 virus [14] and Newcastle disease virus [16], the expression of the M protein alone was insufficient for VLP production in simian virus type 5 [15] and avian pneumovirus type C [18]. The use of recombinant HMPV protein expression to drive the formation of similar HMPV VLPs can potentially overcome the problems associated with the poor cultivation of HMPV in tissue culture. In addition, by direct cloning of the virus genes from clinical material the expression of gene sequences that have not been subjected to extensive tissue culture adaptation can be examined. This therefore affords a relatively simple experimental system with which to examine HMPV morphogenesis. In this study we have examined the capacity of the HMPV F, G and M proteins to form VLPs in mammalian cells, and to further examine the minimal virus protein requirements that lead to VLP formation.

## Results and discussion

### Expression of the HMPV F, M and G proteins in mammalian cells

**Figure 1:**
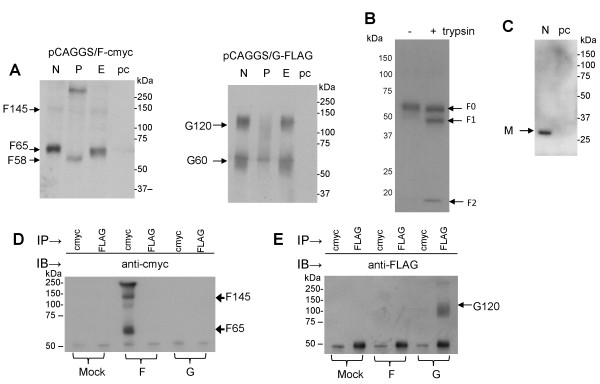
**Construction and surface expression of the F and G gene of HMPV isolate SIN06-NTU271. (A)** pCAGGS/F-cmyc and pCAGGS/G-FLAG were surface-biotinylated and examined by immunoprecipitation with either anti-cmyc or anti-FLAG respectively. Each surface-labelled protein was treated with PNGasF (P), EndoH (E) or non-treated (N). Mock transfected pCAGGS (pc) vector was used to determine specificity of the two antibodies. **(B)** pCAGGS/F-cmyc transfected 293T cells were either non-treated or treated with 0.5 μg/ml TPCK-trypsin for 1 hr at 37°C. The cells were surface-biotinylated and immunoprecipitated (IP) using anti-cmyc **(C)** Cells transfected with pCAGGS/M and immunoblotted using anti-M. **(D** and **E)** Cells singly transfected with pCAGGS/F-cmyc (F), pCAGGS/G-FLAG (G) or pCAGGS (Mock) transfected 293T cells were immunoprecipitated (IP) with either anti-cmyc (cmyc) or anti-FLAG (FLAG) antibodies and Immunoblotted (IB) with **(D)** anti-cmyc or **(E)** anti-FLAG.

The HMPV F and G genes were cloned directly from HMPV strain SIN06-NTU271 and the M gene from SIN05-NTU84 [19] without prior passage of the virus in tissue culture. A cmyc or FLAG tag epitope was placed on the C-terminus of the F and G proteins respectively to generate pCAGGS/F-cmyc and pCAGGS/G-FLAG respectively. The M gene was cloned without an epitope tag to generate pCAGGS/M. The HMPV strain SIN06-NTU271 and SIN05-NTU84 were identified on the basis of sequence analysis as being of the A subtype. 293T cells transfected with either pCAGGS, pCAGGS/F and pCAGGS/G were surface-biotinylated and immunoprecipitated using either anti-cmyc or anti-FLAG antibodies (Figure 1A). The F protein migrated as a 65 kDa protein species (F65), which is the expected size for the uncleaved HMPV F protein [20,21]. An oligomeric F protein species was also detected (Figure 1B, highlighted by F145) which has also been reported previously [8,20]. The glycans attached to the F protein were resistant to Endo-H-treatment, but PNGaseF-treatment resulted in the formation of a 58 kDa protein species (F58), the expected size for the deglycosylated F protein. TPCK-trypsin treatment of the recombinant F protein yielded the presence of a protein species corresponding in size to the F1 protein subunit (Figure 1B), in a similar manner to that described previously [8]. Collectively these data are consistent with the authentic expression of the F protein.

In pCAGGS/G-FLAG transfected cells the G protein migrated as two relatively broad Endo-H-resistant protein bands of approximately 60 kDa (G60) and 120 kDa (G120) (Figure 1A). The size of G60 was consistent with monomeric G protein, while the GA120 kDa protein species was consistent with an oligomeric form of G protein. Several studies have demonstrated the propensity of the HMPV G protein to form oligomers when analysed by immunoblotting. Both G60 and G120 were Endo-H resistant, however following PNGaseF treatment the G protein was reduced to a faint protein smear. This indicated removal of the N-linked glycans, and the residual protein smear was presumably due to heterogeneity of the remaining attached O-linked glycans [7]. The glycosylation analysis was consistent with the presence of mature forms of the surface-expressed F and G proteins. The presence of unusually high levels G protein oligomers in SDS PAGE analysis has been reported previously in avian metapneumovirus [22,23]. Due to its location beneath the plasma membrane, as expected we failed to detect the presence of biotinylated M protein. Immunoblotting of lysates prepared from pCAGGS/M transfected cells with MAbM3F8 (anti-M) revealed the presence of a 28 kDa protein, the expected size of the HMPV M protein (Figure 1C).

Lysates prepared from pCAGGS, pCAGGS/G-FLAG or pCAGGS/F-cmyc transfected cells were immunoprecipitated using either anti-cmyc or anti-FLAG and the immunoprecipitates then immunoblotted with either anti-cmyc (Figure 1D) or anti-FLAG (Figure 1E). Only pCAGGS/F-cmyc transfected cells immunoprecipitated and immunoblotted with anti-cmyc showed the presence of F proteins species corresponding in size to the monomeric and oligomeric F protein (Figure 1D). In a similar analysis G120 was only detected in pCAGGS/G-FLAG transfected cells immunoprecipitated and then immunoblotted with anti-FLAG (Figure 1E). This demonstrated the specificity of the immunoprecipitation assay.

### Interaction between the HMPV F and G proteins

**Figure 2:**
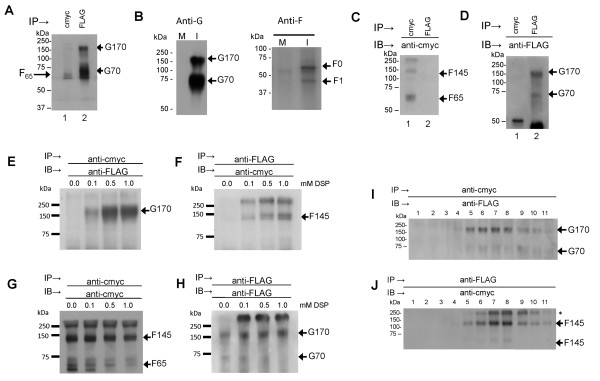
**Stabilization of the co-expressed F and G proteins by *in situ *protein cross-linking. (A)** Cells co-transfected with pCAGGS/F-cmyc and pCAGGS/G-FLAG, surface-biotinylated and lysates immunoprecipitated (IP) with anti-cmyc and or anti-FLAG probed with streptavidin-HRP. **(B)** LLMCK2 cells were either mock-infected or infected with HMPV. At 7 days post-infection the cells were surface biotinylated and lysates were IP with anti-G, or anti-F. The various G and F protein species are indicated. **(C and D)** Cells were co-transfected with pCAGGS/F-cmyc and pCAGGS/G-FLAG, IP with anti-cmyc or anti-FLAG and immunoblotted (IB) with **(C)** anti-cmyc or **(D)** anti-FLAG. **(E**-**H)** Cells were co-transfected with pCAGGS/F-cmyc and pCAGGS/G-FLAG and treated with increasing concentrations of DSP (0-1 mM). Detergent extracts were IP with anti-cmyc and IB with either **(E)** anti-FLAG or **(G)** anti-cmyc, or IP with anti-FLAG and IB with **(F)** anti-cmyc or **(H)** anti-FLAG. **(I and J)** pCAGGS/F-cmyc and pCAGGS/G-FLAG co-transfected cells were treated with 0.1 mM DSP and a detergent extract prepared. This was clarified and loaded onto a 5-30% sucrose gradient. The gradient was fractionated and each fraction **(I)** IP with anti-cmyc and IB with anti-FLAG, or **(J)** IP with anti-FLAG and IB with anti-cmyc. The various monomeric and oligomeric F and G protein species are indicated. High molecular mass F proteins are highlighted with “*”.

Trypsin degradation of the G protein has been previously reported [24], and we have also noted G protein degradation in the presence of quantities of trypsin that are required for F protein activation (Jumat and Sugrue, unpublished observations). Therefore, in all subsequent studies involving G protein expression the trypsin treatment was omitted. Cells were co-transfected with pCAGGS/F-cmyc and pCAGGS/G-FLAG, surface-biotinylated and immunoprecipitated with anti-FLAG. A small but discernible increase in the apparent size of the two G protein species from G60 to G70 and from G120 to G170 was noted (Figure 2A). Similarly, surface-biotinylation of HMPV-infected cells and immunoprecipitated with anti-G showed the presence of G70 and G170 (Figure 2B). This indicated modified processing of the G protein in co-transfected cells, resulting in an apparent molecular mass that is more similar to that observed in HMPV-infected cells. Although this suggested an interaction with the F protein, immunoprecipitation of the co-transfected cells with anti-cmyc or anti-FLAG and then immunoblotted using anti-cmyc (Figure 2C) or anti-FLAG (Figure 2D) failed to demonstrate co-precipitation of these proteins. In a similar manner immunoprecipitation of the F protein from HMPV-infected cells showed the presence of biotinylated species corresponding in size to the F0 and F1 proteins (Figure 2B). Similar ratios of the F0 and F1 proteins were observed following trypsin treatment of the recombinant F protein expressed in 293T cells (Figure 1B).

The F and G proteins are both integral membranes and we hypothesized that the presence of the lipid membrane may be required for stabilization of the protein complex *in situ*. In this scenario the removal the lipid membrane by detergent extraction prior to immunoprecipitation could lead to destabilization of the protein complex. We therefore used Dithiobis [succinimidylpropionate] (DSP) to stabilise the protein complex by *in situ* cross-linking prior to detergent extraction of the virus proteins as described previously [25,26]. Although DSP cross-linked complexes were predicted to be beyond the resolution of SDS-PAGE analysis (>250 kDa), following immunoprecipitation the individual components of the stabilised protein complexes could be released by removal of the covalent cross-links by reductive cleavage using β-mercaptoethanol-treatment.

293T cells were co-transfected with pCAGGS/F-cmyc and pCAGGS/G-FLAG, and the cell monolayers treated with increasing DSP concentrations. Cell lysates were prepared and immunoprecipitated with anti-cmyc, and the presence of the G protein and F protein in the immunoprecipitation assays detected by immunoblotting with anti-FLAG (Figure 2E) and anti-cmyc (Figure 2G) respectively. Immunoblotting with anti-FLAG revealed the appearance of increasing amounts of a 170 kDa FLAG-tagged protein (the expected mass of GA170 protein) with increasing DSP concentrations (Figure 2E). This protein was not present in lysates prepared from non-treated cells. Probing with anti-cmyc revealed similar levels of the monomeric (F65) and oligomeric (F145) F protein species in both non-treated and DSP-treated cell monolayers (Figure 2G).

Similarly lysates immunoprecipitated with anti-FLAG and immunoblotted using anti-cmyc revealed increasing amounts of F145 with increasing DSP concentration, together with a cmyc-tagged protein species >300 kDa (Figure 2F) whose molecular mass was consistent with higher oligomeric forms of the F protein. Probing with anti-FLAG revealed monomeric (G70) and oligomeric forms (G170) of the G protein in both non-treated and DSP-treated monolayers (Figure 2H).

These data suggested that DSP was able to stabilise a protein complex that formed between the F and G proteins. The sedimentation characteristic of cross-linked F and G protein complex was examined using sucrose gradient centrifugation. Cells were co-transfected with pCAGGS/F-cmyc and pCAGGS/G-FLAG and treated with 0.1 mM DSP. A detergent extract was prepared and then applied to a continuous 5-30% (w/v) sucrose gradient. After centrifugation the gradient was fractionated, and the individual fractions immunoprecipitated with anti-cmyc and immunoblotted with anti-FLAG (Figure 2I) or immunoprecipitated with anti-FLAG and immunoblotted with anti-cmyc (Figure 2J). This assay revealed that the co-precipitating F and G proteins were mainly located within fractions 6 to 9, consistent with the co-migration of the DSP stabilized F and G protein complex.

**Figure 3:**
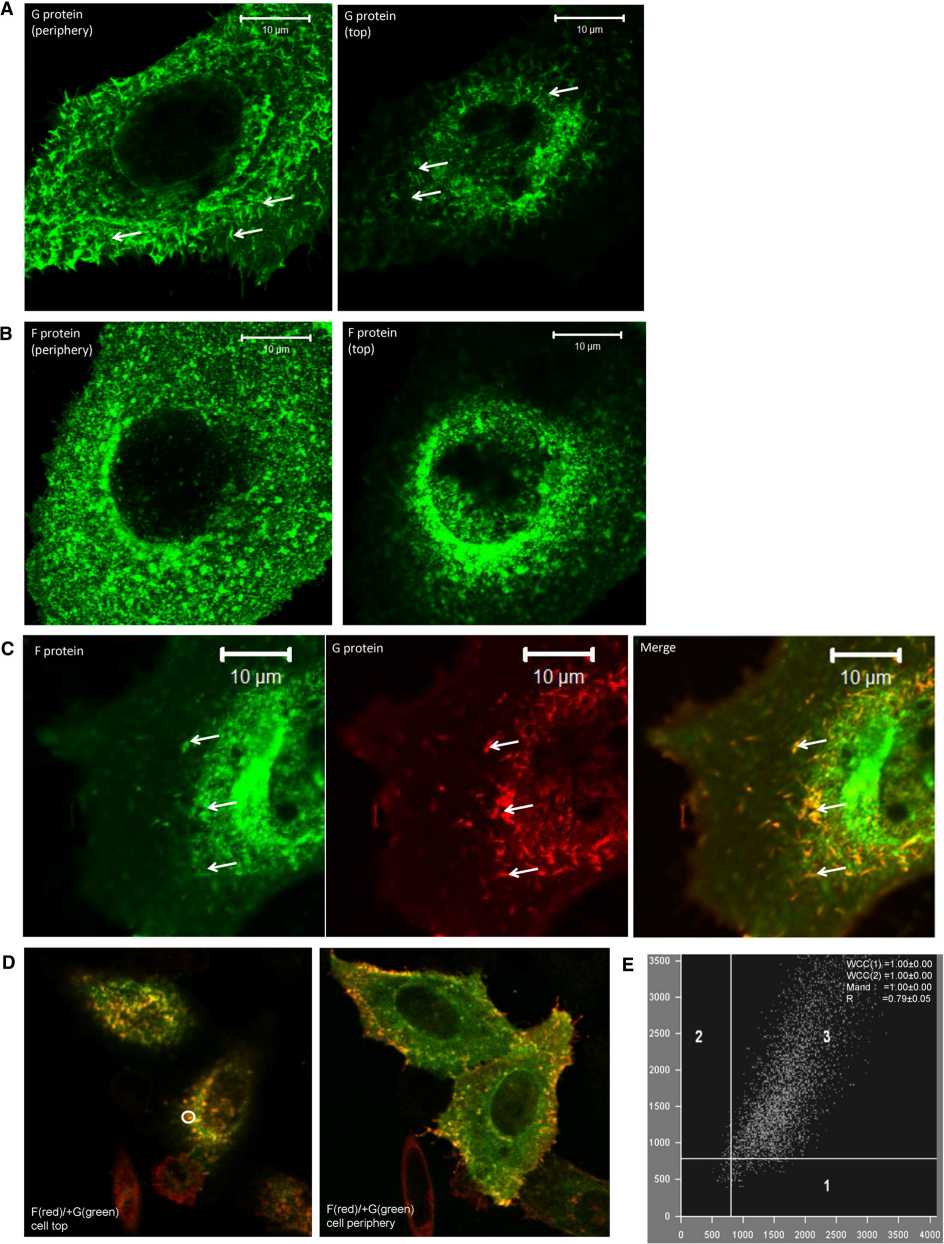
**HEp-2 cells co-expressing F and G proteins showing co-localisation of F and G proteins.** Cells expressing either **(A)** the G protein or **(B)** the F protein were stained using anti-FLAG and anti-cmyc respectively and imaged using confocal microscopy at an optical plane corresponding to the cell periphery and cell top. **(C)** Cells co-expressing the F and G proteins were stained using anti-FLAG and anti-cmyc and imaged using confocal microscopy at an optical plane corresponding to the cell top and cell periphery. **(D)** Co-localisation was calculated using regions (white circles) within the co-transfected cells. WCC(1)-weighted co-localisation coefficient (green channel), WCC(2)-weighted co-localisation coefficient (red channel), Mand- Manders overlap coefficient, R Pearson’s correlation coefficients. **(E)** An example of the scatter-plot region from one of these circles is shown. The x-axis (1) denotes green pixel distribution, the y-axis (2) denotes red pixel distribution and region 3 denotes the yellow pixel combination (co-localising pixels).

We also used fluorescence scanning confocal microscopy (FSCM) to examine the distribution of the G and F proteins in pCAGGS/G-FLAG and pCAGGS/F-cmyc (Figure 3) co-transfected cells. In pCAGGS/G-FLAG transfected cells stained using anti-FLAG a filamentous staining pattern was observed (Figure 3A). pCAGGS/F-cmyc transfected cells stained using anti-cmyc exhibited a more punctate staining pattern (Figure 3B). In cells co-transfected with pCAGGS/G-FLAG and pCAGGS/F-cmyc and stained with anti-cmyc and anti-FLAG a high degree of co-localisation between the F and G proteins within small filamentous projections was observed (Figure 3C). This was supported by statistical analysis using Manders and Pearsons correlation coefficients which showed a high level of co-localisation (Figure 3D and E).

### The HMPV F and G protein complex interacts with the M protein

**Figure 4:**
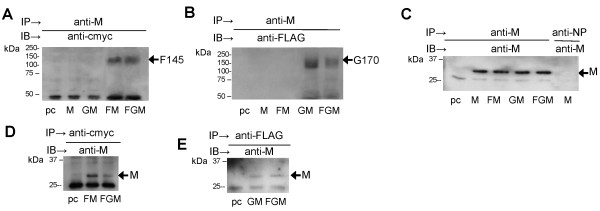
**Evidence that the M protein interacts with the F and G proteins.** Cells expressing the M protein (M), G and M proteins (GM), F and M proteins (FM) or F, G and M proteins (FGM) were immunoprecipitated (IP) with anti-M and immunoblotted (IB) with **(A)** anti-cmyc, **(B)** anti-FLAG or **(C)** anti-M. Cells expressing the F and M proteins (FM) or F, G and M proteins (FGM) were IP with **(D)** anti-cmyc or **(E)** anti-FLAG and IB with anti-M. The various G, F and M protein species are indicated. Cells transfected with pCAGGS are indicated (pc).

We next examined if the F and G proteins could interact with the M protein. Cells were transfected with pCAGGS (pc), pCAGGS/M (M), pCAGGS/G-FLAG and pCAGGS/M (GM), pCAGGS/F-cmyc and pCAGGS/M (FM), or pCAGGS/F-cmyc, pCAGGS/G-FLAG and pCAGGS/M (FGM), and treated with 0.1 mM DSP. Detergent extracts were immunoprecipitated with anti-M and immunoblotted with either anti-cmyc (Figure 4A) or anti-FLAG (Figure 4B). In both cases co-precipitation of oligomeric forms of the F protein (Figure 4A) and G protein (Figure 4B) with the M protein was detected. These immunoprecipitation assays were immunoblotted using anti-M which confirmed the presence of the M protein (Figure 4C). We failed to detect the presence of the M protein in detergent extracts prepared from pCAGGS/M transfected cells that were immunoprecipitated with influenza virus anti-NP (i.e. a non-specific antibody) and immunblotted with anti-M (Figure 4C); confirming the specificity of the M protein immunoprecipitation. The FM- and FGM-transfected cells were immunoprecipitated with anti-cmyc (Figure 4D), and the GM- and FGM-transfected cells immunoprecipitated with anti-FLAG (Figure 4E). Immunoblotting of the immunoprecipitation assays with anti-M revealed the presence of the M protein in each transfection combination. The presence of HMPV proteins in pc-transfected cells were not detected using either antibody. Collectively, these data provided evidence of an interaction between the M protein and the virus glycoproteins.

### Co-expression of the F, G and M proteins lead to the formation of virus-like particles

**Figure 5:**
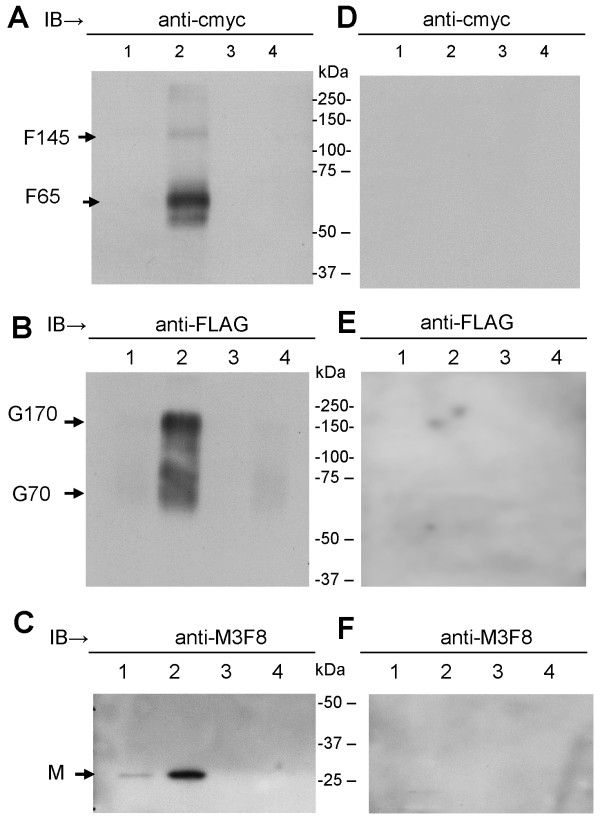
**The co-expression of the F, G and M proteins leads to the formation of virus-like particles (VLPs).** Cells were either transfected with **(A**, **B**, **C)** pCAGGS/F-cmyc, pCAGGS/G-FLAG, pCAGGS/M) or **(D**, **E**, **F)** pCAGGS (mock-transfected) and processed for VLPs as described in Methods. The clarified material was applied to a discontinuous gradient composed of 20% (w/v) sucrose, 50% (w/v) sucrose and 60% (w/v) sucrose. The material at the (1) 0-20%, (2) 20-50% and (3) 50-60% interfaces were harvested, together with the material at the bottom of the centrifuge tube (4). This material was analysed by immunoblotting (IB) with **(A**, **D)** anti-cmyc, **(B**, **E)** anti-FLAG and **(C**, **F)** anti-M. The various G, F and M protein species are indicated.

We hypothesized that the interaction of the HMPV G, F and M proteins could lead to the formation of structures that resembled HMPV particles. These structures would be expected to be cell membrane-bound, and once detached from the cell, they would be expected to have a corresponding buoyant density that could be assessed using density gradient centrifugation. The isolation of HMPV VLPs was achieved based on a modified method for the purification of the related RSV [27]. Cells were transfected with pCAGGS (mock-transfected) or co-transfected with pCAGGS/F-cmyc, pCAGGS/G-FLAG and pCAGGS/M (FGM-transfected) and at 48 h post-transfection the cells were harvested and processed for VLP isolation as described in methods. The clarified cell preparations were loaded onto 10% w/v sucrose cushions, and after centrifugation the resulting pellet subsequently resuspended and loaded onto a discontinuous step sucrose gradient consisting of 20%, 50% and 60% sucrose (w/v) concentrations. After centrifugation the material at the 0-20% (w/v), 20-50% (w/v) and 50-60% (w/v) sucrose interfaces (Additional file 1: Figure S1) was harvested and examined by immunoblotting using anti-cmyc (Figure 5A), anti-FLAG (Figure 5B) and anti-M (Figure 5C). All three HMPV proteins were found to be present at the 20-50% sucrose density interface in FGM-transfected cells, while no HMPV proteins were detected in the discontinuous step sucrose gradient prepared using the mock-transfected cells (Figure 5D, E, F).

**Figure 6:**
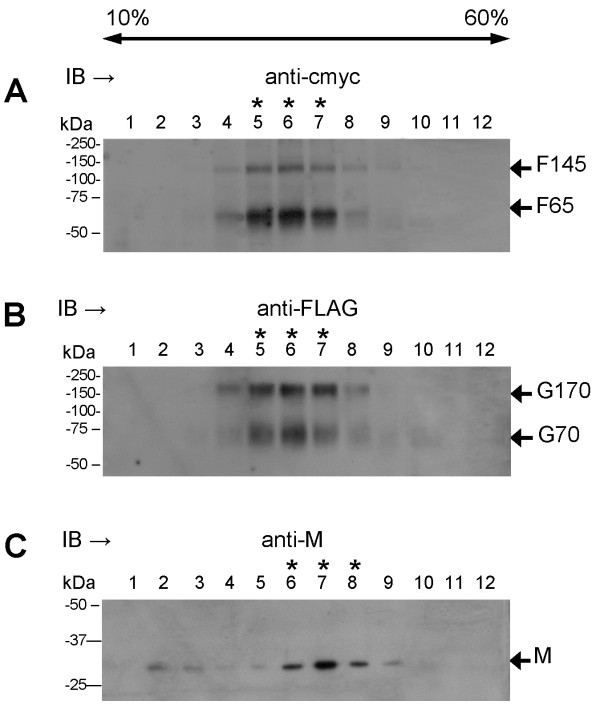
**Continuous sucrose gradient analysis of virus-like particles.** Cells were transfected with pCAGGS/F-cmyc, pCAGGS/G-FLAG, and pCAGGS/M and processed as described for VLP isolation as described in methods. The resuspended pellet from the 10% (w/v) sucrose cushion was applied to a 10-60% (w/v) continuous sucrose gradient. After centrifugation the gradient was fractionated, and each fraction analysed by immunoblotting with **(A)** anti-cmyc, **(B)** anti-FLAG and **(C)** anti-M. The various G, F and M protein species are indicated, as are the peak fractions (*). 1 is the top fraction and 12 the bottom fraction.

In a similar analysis the resuspended pellet recovered from the 10% sucrose cushion was applied to a continuous 10% to 60%(w/v) sucrose gradient. After centrifugation the gradient was fractionated, and the individual fractions proteins examined by immunoblotting using anti-cmyc (Figure 6A), anti-FLAG (Figure 6B) and anti-M (Figure 6C). The F and G proteins were primarily detected in fractions 5 to 7, and this co-migration was consistent with the identification of the protein complex identified above. We noted that while the M protein appeared to co-migrate with the F and G proteins in the continuous gradient, the M protein peak fraction exhibited a slight shift (by 1 fraction), suggesting that the M protein may be associated with more than one type of membrane-bound buoyant particle.

**Figure 7:**
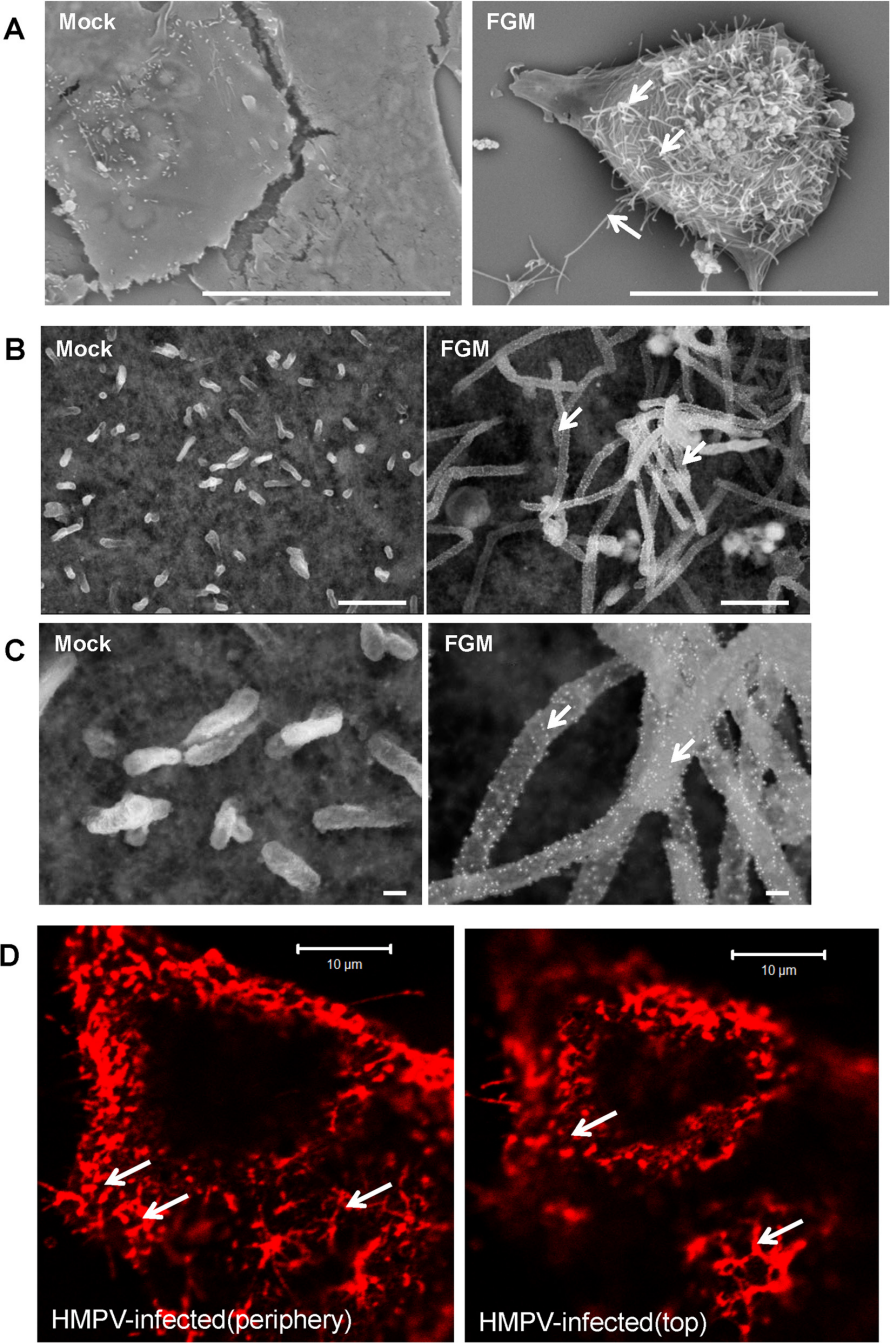
**Visualising HMPV virus-like particles (VLPs) scanning electron microscopy (SEM).** Vero cells were pCAGGS-transfected (Mock) and co-transfected with pCAGGS/F-cmyc, pCAGGS/G-FLAG, pCAGGS/M (FGM) and viewed by **(A)** SEM (magnification × 5,000; bar = 20 μm), the presence of prominent filamentous projections of the surface of FGM-transfected cells is highlighted (white arrows). **(B and C)** Mock-transfected and FGM-transfected cells were stained using anti-FLAG and anti-IgG conjugated to 10nm colloidal gold and viewed under field emission SEM. **(B)** (magnification × 20,000; bar = 1 μm) and **(C)** magnification ×100,000; bar = 100 nm). The distribution of gold particles (white spots) on the filaments is shown (white arrows). The images are superimposed images obtained using the secondary electron scatter and backscatter electron detectors. **(D)** Filamentous projections formed on HMPV-infected cells. At 7 days post-infection HMPV-infected LLCMK2 cells were stained using anti-G and examined at a focal plane corresponding to the cell periphery and cell top. The filamentous staining pattern is indicated (white arrows).

Analysis of mock-transfected and FGM-transfected cells was performed by scanning electron microscopy (SEM). Cells were imaged using conventional SEM and were examined at relatively low magnification. This showed that while mock-transfected cells exhibited a relatively smooth surface morphology at this magnification, FGM-transfected cells showed an increase in a filamentous morphology (Figure 7A). Mock-transfected cells and FGM-transfected cells were examined in more detail using field emission SEM (FESEM) as described previously [28]. Cells were stained using anti-FLAG and anti-mouse IgG conjugated to 10 nm colloidal gold, and the surface morphology and distribution of gold label (indicating G protein distribution) examined. While unlabelled surface features corresponding to microvilli could be detected on the surface of Mock-transfected cells, labelled filamentous projections were detected on the surface of FGM-transfected cells (Figure 7B and C). This was similar in appearance to the filamentous G protein staining pattern exhibited when HMPV-infected cells were stained using anti-G and examined by FSCM (Figure 7D). Collectively, the sedimentation centrifugation and imaging analysis was consistent with the formation of filamentous structures on FGM-transfected cells, which were similar in appearance to the filamentous structures detected on HMPV-infected cells.

### The G protein facilitates VLP formation

**Figure 8:**
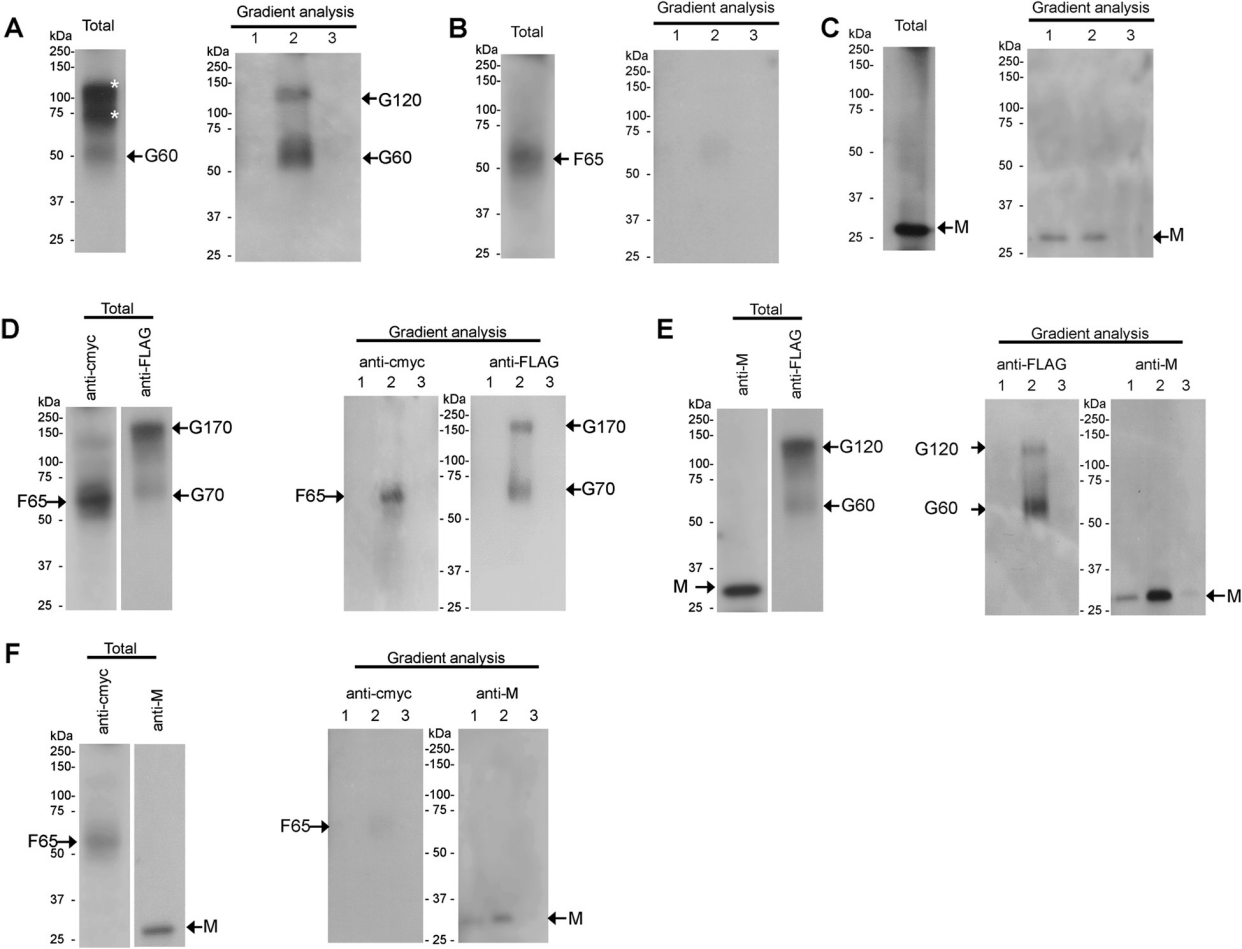
**The HMPV G protein facilitates virus-like particle (VLP) formation by interaction with the F and M proteins.** Cells were transfected with **(A)** pCAGGS/G-FLAG, **(B)** pCAGGS/F-cmyc, **(C)** pCAGGS/M, **(D)** pCAGGS/F-cmyc and pCAGGS/G-FLAG, **(E)** pCAGGS/M and pCAGGS/G-FLAG, and **(F)** pCAGGS/F-cmyc and pCAGGS/M. HMPV protein expression in each transfection was confirmed by analysing the cell lysates by immunoblotting using the appropriate antibody (Total). The transfected cells were processed for VLP formation as described in methods and applied to the discontinuous gradient analysis (Gradient analysis). The material at the (1) 0-20% (w/v) sucrose, (2) 20-50% (w/v) sucrose and (3) 50-60% (w/v) sucrose interfaces was harvested and immunblotted using anti-FLAG, anti-cmyc or anti-M as indicated. High molecular mass G proteins are highlighted by “*”.

The capacity of the individual HMPV proteins to form VLPs was examined using the discontinuous density gradient centrifugation VLP assay described above. In pCAGGS/G-FLAG transfected cells the G protein was detected in the total cell extract, confirming expression (Figure 8A). In the VLP assay immunoblotting with anti-FLAG revealed G protein species of sizes 60 kDa (G60) and 120 kDa (G120) exclusively in the 20-50% (w/v) sucrose interface. In contrast, although F protein expression was detected in pCAGGS/F-cmyc transfected cells, the F protein was not detected in either of the three fractions (Figure 8B) suggesting that it was not efficiently incorporated into the VLP fraction. Similar levels of the M protein were detected in the 0-20% (w/v) and 20-50% (w/v) sucrose interfaces (Figure 8C), suggesting that the M protein was associated with membrane-bound structures with at least two different buoyant densities.

A similar analysis was performed on FG-transfected, GM-transfected, and FM-transfected cells. In FG-transfected cells the presence of the F and G proteins was detected in the cell extract confirming expression (Figure 8D). However, in FG-transfected cells F65 together with G70 and G170 was detected at the 20-50% (w/v) sucrose interface. This indicated that co-expression of the F and G proteins facilitated incorporation of F65 into the VLP fraction. In GM-transfected cells the presence of the M and G proteins were detected in the cell extract confirming expression (Figure 8E). However, a significantly higher level of M protein was detected in the 20-50% (w/v) sucrose interface together with the two G protein species. Interestingly, although both proteins were present within the 20-50% (w/v) sucrose interface we noted no change in the electrophoretic migration of the G protein. In FM-transfected cells the presence of the F and M proteins were detected in the cell extracts (Figure 8F), however the levels of F and M protein in the sucrose interfaces were similar to those detected in cells singly expressing either the F protein (Figure 8B) or M protein (Figure 8C).

## Conclusions

The HMPV F and G proteins have also been shown to bind heparan sulphate [29] and heparin [30] respectively. The HMPV G protein exhibits many similarities to the corresponding RSV G protein e.g. it is heavily modified by O-linked glycosylation and can bind glycosaminoglycans [7,31,32], which suggests a similar role for the HMPV G protein during HMPV infection. Evidence for the interaction between the RSV F and G proteins in the virus envelope [25,33] has provided support for a single protein complex involving both proteins. In this present study we have also provided evidence for the existence of a similar protein complex involving the HMPV F and G proteins. By analogy with RSV, we can hypothesise that such a protein complex will form during HMPV replication.

Our study indicated that co-expression of the HMPV protein was able to form VLPs, and the imaging analysis indicated that the recombinant HMPV particles formed as filamentous structures in a similar manner to that in HMPV-infected cells. Similar virus filaments are formed during morphogenesis of the closely related RSV, suggesting some common features in the assembly process of these different viruses. In this regard we noted that the HMPV G protein facilitated VLP formation independently of other HMPV proteins. Our data also indicated that the interaction of the G protein and the M and F proteins facilitated the incorporation of the latter into these structures. Although the M protein has been proposed to play a role in RSV morphogenesis, recent data suggests that it does not play a role in the initiation of virus particle formation [34]. Our data suggested that expression of the G protein in the absence of other virus proteins is sufficient to form these structures, and its interaction with other virus (and possibly other unknown cell factors) may lead to their active recruitment into VLPs. In particular, our data suggested the presence of the F protein in these structures is largely dependent on its association with the G protein.

It is likely that there are significant differences in the homeostasis in virus-infected and transfected cells which can influence virus processes. Although the VLPs and virus particles are similar in appearance it is also likely that there will be subtle differences between the virus architecture in VLPs and mature virus particles that formed on infected cells [35]. However, given these caveats, we noted an overall similar morphology in the HMPV VLPs and HMPV particles. Given the technical difficulties in growing HMPV in mammalian tissue culture cells (in particular low passage clinical isolates), the generation of HMPV VLPs in tissue culture cells may form the basis of an experimental system to examine aspects of the HMPV maturation process, in particular using protein sequences derived from non-tissue culture adapted viruses). These VLPs may also be a potential source of particulate virus antigen that can form the basis of a vaccine candidate, and future studies will examine these possibilities.

## Materials and methods

### Cloning of the HMPV F, G and M genes from nasopharyngeal washings

Nasopharyngeal washing were collected from children admitted to KK Women’s and Children’s Hospital for respiratory infection as described previously [19]. The F, G and M genes were amplified from the HMPV A2 positive Nasopharyngeal washings (SIN06-NTU271) using the primers F271pCAGGf F271pCAGGmycr, G271pCAGGf G271pCAGGflagr, M84pCAGGf and M84pCAGGr (Additional file 2: Table S1). The PCR products were then inserted into the vector pCAGGS [17].

### Antibodies

The anti-FLAG and anti-cmyc were purchased from Sigma Aldrich and Cell Signalling respectively. M3F8 (anti-M) was prepared from bacterially expressed HMPV M protein. The HMPV G (AT1) and F proteins (Mab58) were obtained from Geoff Toms [36].

### HMPV infection

The HMPV A2 stain NCL03-4/174 was used in the LLC-MK2 cell line in DMEM (BSA, 0.5 μgml TPCK-trypsin) at 37°C for 7 days.

### Expression of F, G and M proteins in (HEK) 293T cells

Cells were transfected using Lipofectamine 2000 reagent (Invitrogen, USA) following the manufacturer’s instructions. The media was changed after 4 hr and the cells were incubated at 37°C for 48 hr before further analysis.

### Western blotting

The proteins were separated by SDS–PAGE, transferred onto PVDF membranes (Immobilon-P, Milipore, USA) as described previously. The tagged proteins were detected with rabbit anti-FLAG antibodies (Sigma-Aldrich, USA), mouse anti-cmyc antibodies (Cell Signaling Technology, USA) or mouse anti-M antibodies (Nanyang Technological University) as appropriate. Protein bands were visualised using the ECL system (GE Healthcare, USA). Molecular masses were estimated using Kaleidoscope markers (Biorad, USA).

### Immunoprecipitation

Cell extracts were prepared at 4°C using RIP buffer (1% NP-40, 0.1% SDS, 150 mM NaCl, 1 mM EDTA, 2 mM PMSF, 2 mM lysine, 20 mM Tris–HCl, pH7.5) and clarified by centrifugation (13,000 g, 10 min 4°C) and immunoprecipitated as described previously [25] using appropriate antibodies. The immunoprecipitation assays were separated using SDS-PAGE.

### Surface labelling

Cells were surface-labelled using EZ-Link Sulfo-NHS-LC-LC-Biotin (Pierce Biotechnology, USA) as described previously [25]. Briefly, cell monolayers were incubated in 0.5 mg/ml solution of EZ-Link Sulfo-NHS-LC-LC-Biotin (Pierce Biotechnology, USA) in PBS pH 8 for 1 hr at room temperature. The lysates were immunoprecipitated with either anti-cymc or anti-FLAG as appropriate.

### Protein cross-linking

*In situ* cross-linking was performed using Dithiobis[succinimidylpropionate] (DSP) (Pierce Biotechnology, USA) as described previously [25]. Briefly, the cell monolayers were treated with between 0.0 and 1.0 mM DSP (100 mM stock solution in DMSO) in PBS pH 8.0, and the cells were incubated at room temperature for 1 hour. The monolayers were then washed with PBS pH 8.0 containing 2 mM lysine prior to detergent extraction with RIP buffer.

### Endoglycosidase digestion

Transfected cells were lysed at 100°C for 10 min in denaturation buffer (0 · 5% SDS, 40 mM DTT). The samples were then made up to a final concentration of either 50 mM sodium phosphate + 1% NP-40 (pH7 5) or 50 mM sodium citrate (pH5 5) and incubated at 37°C overnight with 1000 U PNGase F (New England Biolabs, USA) or 1000 U Endo^_^H (New England Biolabs, USA) respectively.

### Density gradient centrifugation analysis of crosslinked protein complexes

All steps were performed at 4°C. Cell monolayers were extracted using RIP buffer, and the resulting lysate clarified by centrifugation (13,000 g, 10 min). The clarified lysate was layered onto a 5–30% sucrose (w/v) gradient prepared in TEN buffer (1 mM EDTA, 100 mM NaCl, 10 mM Tris-Cl pH 8 + 0.1% Triton X-100). The gradient was centrifuged for 18 hr at 150,000 g and 4°C (Hitachi CP90WX preparative ultracentrifuge; Hitachi Co Ltd, Japan).

### Isolation of VLPs

Cell suspension was subjected to freeze-thaw by ethanol-dry ice and a 37°C water bath. After 3 rounds of freeze-thaw, the cell suspension was clarified (2,500 g for 10 min) and loaded onto a sucrose cushion (10% w/v sucrose in TEN buffer), and centrifuged at 200,000 g for 1 hr at 4°C (Hitachi CP90WX ultracentrifuge). The resulting pellet was resuspended in 200 μl of TEN buffer and loaded onto a discontinuous sucrose gradient (20%, 50% and 60% sucrose (w/v) in TEN buffer). The material was harvested from each sucrose interface was harvested for further analysis. For the continuous centrifugation analysis the sucrose pellet harvested from the sucrose cushion was resuspended in TEN buffer and applied to a continuous gradient (10% to 60% sucrose (w/v) in TEN buffer) and centrifuged at 200,000 g for 18 hr at 4°C. The gradient was harvested by removing 1ml fractions, which were then analysed further.

### Immunofluorescence and confocal microscopy

Cells transfected overnight on 10 mm glass coverslips were fixed with methanol:acetone (1:1) for 15 min at 4°C. The cells were labelled using anti-cmyc, anti-FLAG or anti-M antibodies and the appropriate secondary antibodies (conjugated to either FITC or Alexa Fluor 555) as described previously [37]. The cells were visualized using a Zeiss Axioplan 2 confocal microscope using appropriate machine settings

### Scanning electron microscopy (SEM)

Cells were processed as described previously [28]. Briefly, transfected cells were fixed using 0.1% glutaraldehyde and labeled using anti-FLAG and anti-rabbit IgG (1/100 dilution) conjugated with colloidal gold (10 nm) (Sigma-Aldrich, USA) for 4 hr at room temperature. The cells were then post-fixed in 2.5% glutaraldehyde and 1% OsO_4_ prior to critical point drying. The cells were carbon coated and viewed using either a Jeol 5600 or a Jeol FE-SEM7000 using appropriate machine settings.

## Competing interests

The authors declare that they have no competing interests.

## Authors’ contributions

LLH performed cloning and analysis of the recombinant F and G proteins. MRJ performed analysis of virus-infected cells. YF and TCA produced anti-M and characterisation of recombinant M proteins. PSW performed scanning electron microscopy. NWST participated in design and coordination. BHT performed electron microscopy and participated in its design and coordination. RJS conceived the study and participated in its design and coordination. All authors read and approved the final manuscript.

## Supplementary Material

Additional file 1: Figure S1 Discontinuous sucrose gradient concentration of virus-like particles from 293T cells. Cells were processed for discontinuous density gradient centrifugation as described in text. After centrifugation the opalescent bands at each interface in the sucrose gradient was detected using a focused light. Image of ultracentrifuge tube shows the presence of opalescent bands at the (1) 0-20% (w/v) sucrose, (2) 20-50% (w/v) sucrose and (3) 50-60% (w/v) sucrose interfaces in the analysis from pCAGGS/F-cmyc, pCAGGS/G-FLAG, pCAGGS/M transfected cells is shown. The opalescent band at interface is highlighted (black arrow). Click here for file

Additional file 2: Table S1 Primers used for cloning of F, G and M genes into pCAGGS vector. The underlined bases indicate restriction enzyme recognition sites. The bases in italics are those coding for cmyc or FLAG tags. The GenBank/EMBL/DDBJ accession numbers for the genome sequences of HMPV isolates SIN06-NTU271 F and G genes and SIN05-NTU84 M gene are EF397627, JQ309677, JQ309649 respectively. Click here for file
