# Supplementary figures and images for: Evidence for the interaction of the human metapneumovirus G and F proteins during virus-like particle formation
Source: Virol J. 2013 Sep 25;10:294. doi: 10.1186/1743-422X-10-294 (PMC3849350; doi:10.1186/1743-422X-10-294)

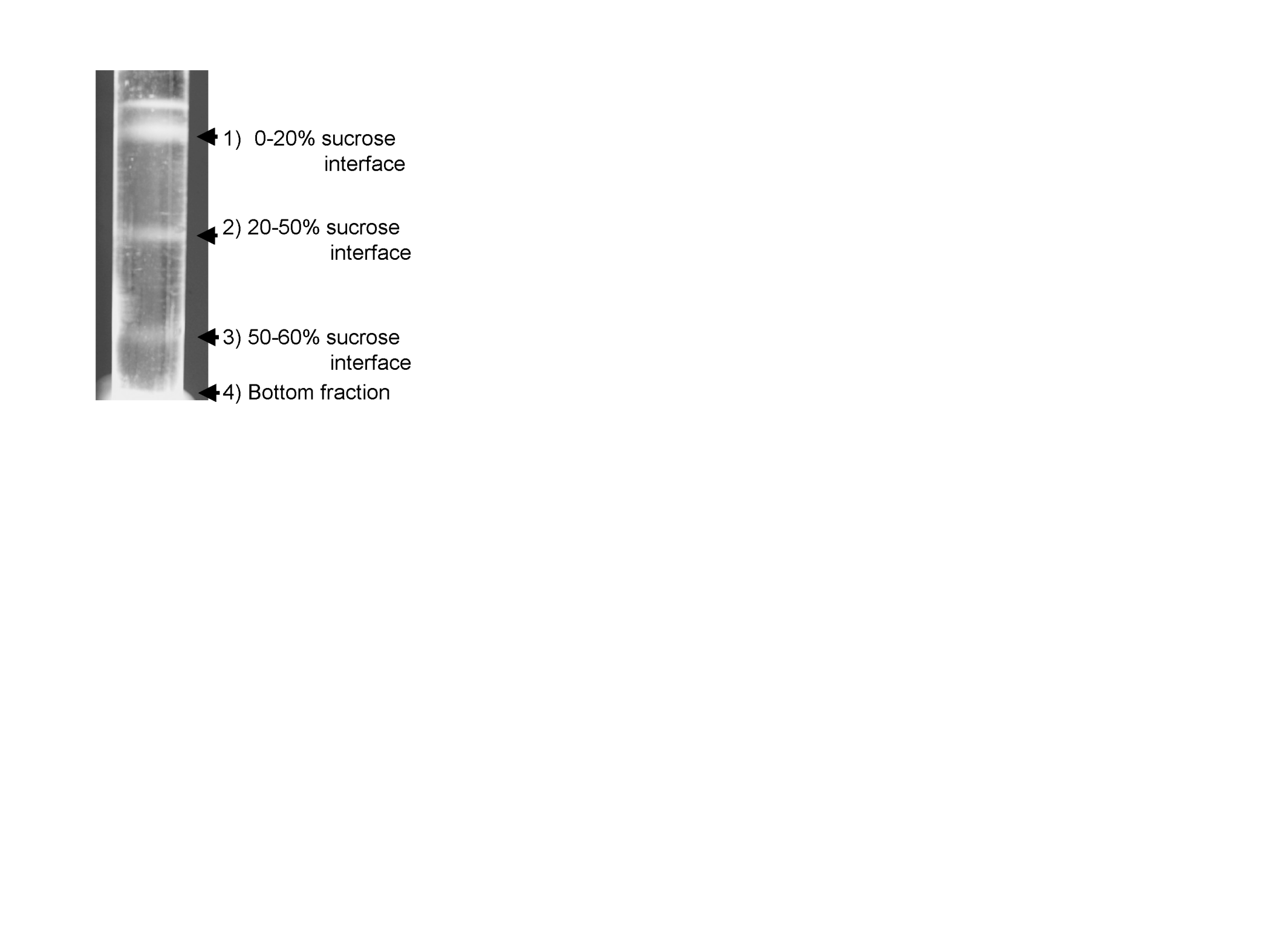

Supplement: Additional file 1: Figure S1 — Discontinuous sucrose gradient concentration of virus-like particles from 293T cells. Cells were processed for discontinuous density gradient centrifugation as described in text. After centrifugation the opalescent bands at each interface in the sucrose gradient was detected using a focused light. Image of ultracentrifuge tube shows the presence of opalescent bands at the (1) 0-20% (w/v) sucrose, (2) 20-50% (w/v) sucrose and (3) 50-60% (w/v) sucrose interfaces in the analysis from pCAGGS/F-cmyc, pCAGGS/G-FLAG, pCAGGS/M transfected cells is shown. The opalescent band at interface is highlighted (black arrow). [file 1743-422X-10-294-S1.tiff]
