# Supplementary material for: Evidence for the interaction of the human metapneumovirus G and F proteins during virus-like particle formation
Source: Virol J. 2013 Sep 25;10:294. doi: 10.1186/1743-422X-10-294 (PMC3849350; doi:10.1186/1743-422X-10-294)
Supplement: Additional file 2: Table S1 — Primers used for cloning of F, G and M genes into pCAGGS vector. The underlined bases indicate restriction enzyme recognition sites. The bases in italics are those coding for cmyc or FLAG tags. The GenBank/EMBL/DDBJ accession numbers for the genome sequences of HMPV isolates SIN06-NTU271 F and G genes and SIN05-NTU84 M gene are EF397627, JQ309677, JQ309649 respectively. [file 1743-422X-10-294-S2.doc]

| F271pCAGGf | (5’-gcggtaccgttatggcttggaaagtggtg-3’), |
| --- | --- |
| F271pCAGGmycr | (5’-gcctcgagcta*cagatcctcttctgagatgagtttttgttc*actgtgcggtatgaa gcc3’), |
| G271pCAGGf | (5’-gcggtaccaccatggaggtgaaagta-3’), |
| G271pCAGGflagr | (5’-gcctcgagcta*tttatcgtcatcgtctttgtaatc*tattgttggtgtgctggt-3’). |
| M84pCAGGf | (5’-gcggtaccattatggagtcctatctg-3’) |
| M84pCAGGr | (5’-gcctcgagttatctggacttcagcac-3’). |
